# Clinical Lecture on Angular Curvature; Its Pathology and Treatment

**Published:** 1877-09

**Authors:** J. F. Miner


ART. II.—*Clinical Lecture on Angular Curvature; Its Pathology and Treatment.* By Prof. J. F. MINER, M. D. Reported by CHAS. A. WALL, B. S.

GENTLEMEN :—At the close of our last clinical lecture, and in connection with the *post-mortem* examination of a patient whose death was due to Pott's disease, we were about to consider the anatomical changes which occur in angular curvature, with a view to answering the question, whether they are such as admit of partial, or total restoration to the natural form. In inflammation of the cancellated structure of bone, we notice fullness of the vessels, and general infiltration of plastic material. The connective tissue swells and becomes filled with a gelatinous or fatty substance and in the same ratio the bony structure becomes rarefied by either change or absorption. This change has received by Rokistanskey the name of osteo-porosis. If the inflammation progresses farther, the cellular bodies are converted into parental pus cells, which burst and diffuse the embryonal pus cells through the entire structure. If diffused, this constitutes what is called *purulent infiltration;* if the inflammation is acute and circumscribed, it gives rise to an abscess. In general inflammation of bone (*endostitis*) the bone becomes soft and pliable as in rachitis, and even more so. This condition of bone must be constantly remembered in estimating the restoration we may obtain in deformity. The recovery of the cancellated structure of bone from inflammation or suppuration is accompanied by the formation of denser osseous material. Now, it is evident that the softened vertebral bodies, cannot sustain, and do not sustain the superincumbent weight without being altered in shape. When the disease is established the bones are altered in shape and the projection observed in the spine shows this sufficiently well. We can remove the weight by placing the patient in recumbent position, but does this exercise any force towards restoring the soft and ulcerated bones to natural form. By this means we may arrest the farther progress of the disease, but it is not very obvious that we produce any influence capable of restoring the natural form of the bones; all we can possibly accomplish is the arrest of the deformity; we cannot change the form of the vertebral bodies. If it is impossible to change or restore these bones while soft and pliable, what must be expected when they become consolidated and encrusted by new bony deposits. It seems to me then, that in posterior curvature of the spine, which originates in inflammation of the vertebral bodies, there can be but one opinion:—it is impossible to restore to the natural form: to attempt it, is often to induce incurable disease. Certainly we are unable to correct a deformity that has been caused by so material anatomical changes, and has been rendered permanent by osteophytic bands passing from one part of the spine to another, fixing it in a new, but unalterable position. As to softening of the intervertebral fibro-cartilages, the facts are not so well determined. It seems probable that this structure may become the seat of inflammatory softening, and clinical cases are not wanting which appear to be of this nature. The local symptoms were not severe, suppuration never occuring, and recovery though slow yet nearly perfect. Dr. Beauer says that such were the cases in which he succeeded in slightly reducing the posterior curvatures, but was never successful in completely restoring one single case. Considering that the flexibility of the spine chiefly rests *with* the intervertebral cartilages, and that the inflammatory process renders their structure still more soft and yielding, it would seem that this was the only form of posterior curvature which can at all be regarded as capable of positive cure. Possibly such disease has simulated so closely the true change in the bone and having recovered or partially recovered, has misled as to curability of posterior curvature.

Dr. Markoe of N.Y., says, "that true tubercle may exist in bone, I believe is denied by few pathologists, that it is a common affection is denied by many." I have wearied you thus long, upon the questions of curability, but it is well to know the extent of our usefulness, when if we go much farther, we soon enter the wide domain of pretension.

We come now to consider a moment the *causes* of this disease. It has been said that surgeons almost without exception have looked upon Pott's disease as caries of the spinal bones, arising from constitutional causes—from consumption or syphilis, and have overlooked the influence which traumatic injuries would be likely to exert, such as falls, twists, concussions, &c., &c. admitting the constitutional causes with remarkable readiness, and underrating the effects of injuries. The general belief prevails, that posterior curvature is the attendant of poverty, neglect and diseased deteriorated constitutions. It is of the utmost importance that this question of cause, be settled in accordance with the exact truth, for upon it we base our expectation and direct our means of cure. If we accept the long cherished view, that it arises in scrofulous and diseased children from constitutional causes we at once commence our Cod Liver Oil, Iod. of Potass, Bark and Iron plan of medication and allow the spinal deviation to take care of itself. If we adopt the idea that it more often arises from traumatic causes we apply local remedies, and entertain expectation of greater or less relief. The more fully we can adopt this belief the more useful we shall become to our patients, preventing difficulties which, once established cannot be cured.

At the present time, the best informed surgeons are ready to admit the effects of local injuries, generally deny the assumption of posterior curve occurring in the poorly fed and poorly cared for diseased children of low life—find it more often in the higher classes, and where there can be no trace of constitutional disease acting as a cause—when indeed we have so healthy and vigorous constitution that nature overcomes and restores from a condition of most extensive disease. It is almost absurd to suppose that recovery could be obtained from the natural efforts of a system so diseased as to develop the malady it is at the same time able to cure. When we remember the effects of trifling injuries to the spine, the anatomical condition in early life, so unlike that of adults, and the various strains, concussions and other injuries so liable in the ramblings of children, we *cannot* exclude these, but must regard them as most operative and important in the production of inflammation, softening and ulceration of the vertebral bones, which is the cause of spinal posterior curve. That other causes are liable to lead to similar results cannot be denied. Sudden checking of profuse perspiration is as apt to give rise to periostitis of the vertebral as to other bones of the system. Hooping cough is said to be the cause of the disease, and if so, probably acts more as strain or injury than in any other manner. Syphilis also, no doubt, in rare instances, attacks the vertebræ and the intervertebral cartilages, but beyond all comparison the most frequent cause of posterior curve will be found in traumatic injuries,—injuries which are slight and at the time attract little attention. The time and nature of the injury may not be known; generally the disease appears to spring up spontaneously and makes considerable progress before it attracts any attention.

It may be well before closing our remarks upon the causation of posterior deformity, to acknowledge constitutional causes as adequate to the production of the disease. Bad hygiene and deranged and imperfect nutrition, scrofulous diathesis and all other conditions of debility, are predisposing causes, at least, and are adequate in extreme cases, alone to cause it.

*Symptoms.* This disease is of slow growth, a period of weeks or months may intervene between the cause and the final appearance of curve; the symptoms attending this period are obscure and of a general nature, the disease may exist for some time without being noticed, so little inconvenience is occasioned by it. There is generally inactivity, debility, anæmia, loss of appetite, turbid urine, &c., &c. These little patients neglect the sports of their play-fellows, seek for retirement, and lay down assigning shortness of breath, palpitation or pain in the bowels, perhaps, as the cause. These preliminary symptoms are present for an indefinite period sometimes but a few days at others for several weeks or months.

The severe cases sooner manifest the positive signs, *pain, stiffness of the spine,* and the *protrusion of one or more spinous processes.* Other and more serious signs, or rather effects of this disease supervene, such as great emaciation, paralysis or contraction of the muscles of the thigh, abscess, malformation of the chest, &c., &c. The cause of the emaciation is not well known, and in some instances is not present. The paralysis of the lower extremities has been erroneously ascribed to mechanical compression of the spinal cord. Clinical observation and post-mortem examinations have not justified this view, but have disclosed the real cause. The spinal cord is suspended in such manner within the spinal canal as to be uninjured by the numerous changes and postures of the body. The foramina for the transmission of the spinal nerves are much larger than required so that pressure is not likely to be produced. Rare cases only have been found where effusion or forming of abscess within the spinal canal gave rise to pressure, rarer still where sequestrum had formed and become detached so as to press upon the spinal cord. The *true cause* consists in hyperæmia, irritation or inflammation of the membranes or of the cord itself.

This is supported by negative and positive clinical facts. Paralysis seems entirely independent of the degree of deformity, makes its appearance in the earlier and more acute stage and often passes off at a time of the greatest deformity. Rest in recumbent position, and cold applications to the spine seems to relieve it, at any rate it disappears while the causes of pressure are unrelieved. Again the paralysis is generally limited to the motor fibers alone. This is explained by these being in the anterior part of the spinal cord, and nearer to the seat of the disease more exposed to inflammation or irritation. Paralysis is rarely, if ever, present when the active symptoms of the disease have entirely disappeared. We have recently dismissed a case from the Hospital, now *well,* and active as a Grocer's Clerk, which for a year after admission was almost perfectly paralyized in the lower extremities.

*The contraction of the muscles* of the thigh is sometimes mistaken for hip-joint disease and treated accordingly. It is caused by irritation of the spinal cord and from the same pathological conditions as the paralysis.

*Abscess.* If the disease of the vertebral or the adjacent tissues advances to ulceration, the formation of consecutive abscess is the result. These make their appearance remote from the seat of the disease, and since unattended by pain or discoloration of integument are sometimes called cold abscess. They also receive other names from the regions in which they appear. *Psoas* abscess is when the pus passes over the psoas muscles and points in the thigh below pouparts ligament. Similar abscesses are observed in various other regions as the neck, axilla, chest; (rarely) sometimes in the pelvis, and discharge through the rectum. These regions are determined mainly by the part of the spine affected with caries, though the windings of pus in its escape is often quite remarkable.

*Treatment.* All the fluctuations which have taken place as to causes of scrofulosis have influenced the treatment of posterior curvature. When the blood was regarded as contaminated by a peculiar dyscrasic agent, its illimination through the secretory and excretory organs, was laid down as the indication for cure, this was the *humoral* pathology; the *solidarists* contended that if the nutritive fluid of the body was thus contaminated, the morbid principle would certainly be incorporated in the solid structures. Other schools adopted other theories and pursued other plans of treatment shifting from one absurdity to another, vainly seeking for relief in means which were rarely useful often barbarous and prejudicial. Unfortunately the barbarism of former periods is still continued in the local treatment, and moxa, issue and the like derivatory remedies are often, perhaps most generally resorted to. As this is done by regular and educated physicians, it is well to consider a plan of treatment which is either rational or irrational and must stand upon its own merits. If it was only advised by quacks and imposters no mention need be made of it. This treatment has grown out of wrong views in pathology but in no way commends itself even admitting the tubercular or dyscrasic condition of the system to be the cause, for it must now be obvious to the crudest observer that issues, setons, moxa and the hot iron are absurd, cruel and injurious appliances for the removal of tubercular matter. The advocates of this local treatment seem to repose but little confidence in these appliances generally in tuberculosis, not having attempted to put them in requisition when the lungs or other organs are invaded; this inconsistency is the strongest condemnation of the plan. And if clinical fact were obtained to prove their efficacy they would tend to disprove, rather than substantiate the theory. Such facts do not exist except in imagination.

We are told that local derivation is less designed against tubercular deposits themselves, than against the process that initiates them. The idea is that some sort of inflammation precedes or initiates tubercular deposits which might be mitigated and the deposits prevented by early establishing a more intense inflammation in contiguous but less important structures. Superficial reasoning like this may seem plausible at first, but a little more reflection discloses the fallacy. We cannot detect the first appearances of this disease; it is rarely if ever presented for treatment until the so-called tubercular deposit has taken place and produced its characteristic degeneration. Again it is against all physiological law to drive disease from one structure to another by the same nerves and vessels; it would seem that the disease would be thus directly stimulated. Besides all physiological objections to derivation, they are so severe as seriously to affect the constitution, creating irritative fever, disturbing rest and appetite, and producing drain upon the system already taxed and enfeebled beyond endurance. Again such local appliances interfere with the supine posture, so indispensable in incipient cases of posterior curve. As we deny the tubercular origin of this disease, the question remains whether derivation can possibly benefit the existing morbid condition, of which posterior curvature is but a symptom. Is there any rational theory to sustain belief in such curative measures? will softened ulcerated bone recover the sooner for any drain which may still further reduce the system? Does it appear to any physician that fractured or otherwise diseased bone recovers sooner or is more perfectly restored by application of blisters and setons in the near vicinity? has not counter-irritation been tried shamefully long for the cure of tubercular disease of the lungs and is it not now abandoned by all well balanced thinking physicians? What counter-irritation or derivation can have to do with restoring the normal anatomical conditions cannot be conceived; the grossest ignorance in pathology alone, can tolerate such absurdity. The practice of counter-irritation has given no satisfaction to either surgeon or patient and should be abandoned so far as joint, bone and spine are concerned, and I need not withhold my opinion that the same is true of every other disease for which it has been, and still is so frequently adopted. I have dwelt upon this point, for I desire to impress you if possible with the conviction that this barbarous unscientific distressing and injurious system of treatment will never yield you any results but disappointment, chagrin and failure and generally subject you to the disgust and contempt of the little sufferers who would otherwise regard your coming with joy.

REST is the great therapeutic agent in this disease; *rest* and relief of pressure. Since the horizontal position is by far the most effective in relieving pressure and insuring *rest* it must be considered first in its importance. Rest and relief of pressure must not in all stages be obtaind by horizontal position. During the formative and acute stages horizontal position will answer our indications better than anything else, if you are fortunate enough to find cases while the disease is recent, attended with heat, pain, restlessness and general fever. The horizontal position constantly maintained will afford you more relief than any splint however ingeniously it is made and applied. To this in some cases extension may be added, thus more completely relieving the pressure by overcoming muscular contraction and in some degree preventing deformity. This plan is to be continued through the acute stage, indeed through all stages modified by various means. But the time comes, when you must not confine your patient, but give air and exercise, and this brings us to a consideration of some of the other measures calculated to insure rest and relief of pressure. While supine posture, rest, and extension comprise the means of relief, in incipient cases of posterior curve the time comes when supine posture is no longer desirable and when we must give rest and support by mechanical means. You are well aware of the great variety of supports which have been presented to the profession for this purpose, all posessing more or less merit. I am able to show some of them and point out the deficiencies and advantages which they possess. The great majority of cases require some of these appliances when they are presented to you, and have passed that acute stage when recumbent posture and complete rest is desirable. In *Consecutive Abscess*, (dorsal, lumbar, psoas and illiac), we advise puncture, and in diffuse abscess, or when the purulent deposit has not become circumscribed we would make free incisions through the fascia and other parts which confine it. Some authors however do not advise opening these abscesses as long as it can be avoided.

In this brief and hasty account of posterior curvature of the spine I have only attempted to outline the disease, guard you against prevalent errors in regard to it and mention some of the more plain and obvious indications for its management and cure.

If I have *succeeded* to impress you with its true character in sufficient degree to prevent your adopting for relief measures both painful and injurious, and have in any good degree drawn your attention to the sensible and safe methods recommended, I have done you some favor, and your patients an inestimable benefit.

